# Are current guidelines adapted for patient eligibility to PrEP? A case report

**DOI:** 10.1186/s12879-019-4239-1

**Published:** 2019-07-10

**Authors:** Elise K. Van Obberghen, Delphine Viard, Alain Lafeuillade, Alexandre Civiletti, Fanny Rocher, Milou-Daniel Drici

**Affiliations:** 1 Centre Régional de Pharmacovigilance de Nice-Alpes-Côte d’Azur, Hôpital de Cimiez - Pavillon Victoria - CS 91179, 06003 Nice cedex 1, France; 2 Service des Maladies Infectieuses, Hôpital Sainte Musse, Toulon, France

**Keywords:** Pre-exposure prophylaxis, Antiretroviral therapy, Men who have sex with men, HIV diagnostic tests

## Abstract

**Background:**

Despite effective antiretroviral therapy developed over the last decade, HIV infection remains a major worldwide public health problem. Recently, a promising preventive treatment has been made available for HIV prophylaxis, PrEP for pre-ExPosure Prophylaxis. Indeed, it was shown to significantly reduce the risk of HIV infection in patients exposed to high risk of infection such as men having sex with men (MSM), heterosexuals and people who inject drugs. Several issues pertaining to PrEP remain uncertain including short and long-term adverse events, drug resistance, risk compensation and resurgence of other sexually transmitted infections.

**Case presentation:**

We report a case of a 52-year-old MSM eligible for PrEP as he was exposed to a high risk of HIV infection, presented no clinical symptoms of HIV primary infection and was seronegative for HIV. PrEP therapy was then initiated with fixed association of emtricitabine-tenofovir disoproxil. One month later, HIV tests using two different assays were positive, despite perfect compliance reported by the patient and confirmed by plasma drug level. A retrospective search for plasma viral RNA in the blood sample before PrEP initiation turned out positive. Genotyping and treatment sensitivity performed on sample after one month of PrEP showed a virus resistance to lamivudine and emtricitabine.

Similar cases in the literature and pivotal studies have reported HIV infections in patients initiating or undergoing PrEP. These patients where either infected but still seronegative, displaying no clinical symptoms upon enrollment, or became infected during PrEP. Reasons are mainly poor compliance to treatment, resistance to PrEP, and lack of diagnosis before PrEP. Guidelines advocate safe sex behavior before initiation, search for clinical signs of HIV primary infection and two different serologic tests performed with one-month interval.

**Discussion and conclusions:**

Our patient newly HIV infected received PrEP as he was still seronegative. Current recommendations fail to screen recently HIV infected, but still seronegative patients who are initiating PrEP. This issue raises strong concerns regarding the lack of adequate selection for eligibility to PrEP and may contribute to exposing partners to HIV infection and select viral mutations. Infection risk could be minimized by search for plasma viral HIV RNA at pre-inclusion, at least for patients suspected of unsafe behaviors such as non-respect of the non-exposure period before PrEP initiation.

## Background

Despite effective therapies developed over the last decade, Human Immunodeficiency Virus (HIV) infection remains a major worldwide health problem. Recently, a promising treatment has been made available for HIV prophylaxis called PrEP (pre-ExPosure Prophylaxis). Indeed, it was shown to significantly reduce the risk of HIV infection in patients exposed to high risk of infection such as men having sex with men (MSM), heterosexuals and people who inject drugs. However, several issues pertaining to PrEP remain uncertain including short and long term adverse events, drug resistance, risk compensation and resurgence of other sexually transmitted infections. We report a case of inadequate PrEP exposure in a patient who was already HIV infected but still seronegative at PrEP initiation.

## Case presentation

A 52 year-old MSM consulted for PrEP prescription. At the initial consultation on the 6th of January, 2017 (M-1), he reported high risk behaviour but was asymptomatic and HIV seronegative. He was counselled on the appropriate use of condoms and asked to return for review one month later for follow up HIV testing and to initiate PrEP. He then returned for review the 7th of February, 2017 (M0). At this time he remained asymptomatic and HIV negative (assay performed with 4th generation combined antigen-antibody HIV ELISA test). As the patient was eligible for PrEP he was further counseled and then PrEP was prescribed (daily fixed dose combination of tenofovir disoproxil and emtricitabine, 1 pill per day). Explicit information was provided on a potential contamination risk despite PrEP and on the importance of maintaining a proper use of condoms. Written informed consent was obtained from the patient. On a one-month follow up consultation on the 7th of March, 2017 (M1), HIV tests proved positive using two different immunological assays: HIV Combi PT® and VIH Vidas Duo Biomerieux®. Positivity of plasma HIV RNA (96 323 copies/ml) was assessed as well. Perfect compliance of PrEP intake was reported by the patient and plasma drug levels were consistent with this. PrEP was ceased and a tri-therapy with darunavir ethanolate, ritonavir and dolutegravir was commenced. Then the patient reported to have had sex with 4 partners during the month preceding PrEP initiation and with two others in the month after.

The baseline blood sample (M0) was retested. Fourth generation Ag/Ab test was negative but HIV RNA was positive at 190 copies/mL. Genotyping and treatment sensitivity performed on the (M1) blood sample revealed virus resistance to both lamivudine and emtricitabine (Mutation M184I), but not to tenofovir. Phenotype and sensitivity tests showed a partial efficacy of PrEP suggesting possible contamination with a virus already presenting M184I mutation.

## Discussion and conclusion

PrEP is an effective preventive treatment for HIV infection that has been shown to provide a relative risk reduction of 43% [1, 2]. Marketing authorization for PrEP was granted with an indication restricted to MSM under specific conditions of eligibility (based on the Morlat Report, 2016), for a fixed-dose combination of emtricitabine and tenofovir disoproxil fumarate on a chronic intake basis. Despite these recommendations that were strictly applied, our patient was already infected when exposed to PrEP.

Similar cases in the literature and pivotal studies have reported HIV infections in patients initiating or undergoing PrEP. These patients where either infected but still seronegative, displaying no clinical symptoms upon enrollment, or became infected during PrEP.

Seroconversion after PrEP initiation usually results from poor compliance to treatment, and can lead to drug resistance [3, 4], which was not the case for our patient. Since plasma viral RNA was already present in the baseline sample which was still seronegative for HIV, we estimate a probable contamination less than 10 days before the sample date [5]. Unfortunately it was not possible to obtain a genotype from the specimen taken at (M0). Therefore it is not possible to know if the patient was infected with a resistant virus, or resistance was developed whilst on PrEP.

This case report highlights the importance of maintaining condom use before PrEP initiation and under PrEP. Although abstinence or use of condoms is essential for preventing HIV infection, some patients aware of its importance find it difficult to maintain proper use of condoms. Its absolute use cannot be verified at time of inclusion, therefore its appreciation solely relies upon patient reporting. Consequently very high risk patients presenting difficulty in maintaining condom use are more at risk of being infected prior to PrEP initiation. Therefore, clinical examination and HIV serology may not accurately reflect the infection real status at the time of PrEP initiation, as in the case of our patient.

Current recommendations fail to screen the recently HIV infected but still seronegative patients entering the program. Those patients should not be eligible for PrEP as it may lead to a risk of under diagnosis, a selection of viral mutations, conferring resistance to emtricitabine – tenofovir, facilitating the dissemination of drug-resistant viruses, and possible contamination of sexual partners. Empirical prescription of one or two active agents added to the existing PrEP in patients for whom there is a doubt on their abstinence or use of condom may protect sex partners and decrease resistance. However, this must be evaluated in well-conducted clinical trials. In the reported studies on PrEP, the rates of infection at baseline are small, a mere 0.72% in the PROUD study [6]. Although the number of patients in this particular situation is low, it is difficult to expand HIV RNA testing on a routine basis as the cost is not negligible. Therefore, a systematic search at pre-inclusion for plasma viral HIV RNA for very high risk patients could be a solution. Even though it incurs a cost, albeit modest in comparison with the monthly cost of PrEP, this additional test could improve initial screening for eligible patients to PrEP and be weighed against the possibility of avoiding HIV contamination.

Hence reserving plasma viral HIV RNA testing at pre-inclusion for patients who seem to be at very high risk, or not able to strictly adhere to condom use prior to PrEP initiation, might be an answer to improve targeting patients for PrEP. Therefore efforts should be made by healthcare providers to facilitate access to HIV RNA testing at least for patients at very high risk for HIV infection.

## Data Availability

Data sharing is not applicable to this article as no datasets were generated or analysed during the current study.

## Abbreviations

HIV: Human Immunodeficiency Virus
MSM: Men having sex with men
PrEP: pre-ExPosure Prophylaxis prophylaxis

## References

[1] Grant RM, Lama JR, Anderson PL, McMahan V, Liu AY, Vargas L (2010). Preexposure chemoprophylaxis for HIV prevention in men who have sex with men. N Engl J Med.

[2] Molina J-M, Capitant C, Spire B, Pialoux G, Cotte L, Charreau I (2015). On-demand Preexposure prophylaxis in men at high risk for HIV-1 infection. N Engl J Med.

[3] Thigpen MC, Kebaabetswe PM, Paxton LA, Smith DK, Rose CE, Segolodi TM (2012). Antiretroviral Preexposure prophylaxis for heterosexual HIV transmission in Botswana. N Engl J Med.

[4] Knox DC, Anderson PL, Harrigan PR, Tan DHS (2017). Multidrug-resistant HIV-1 infection despite Preexposure prophylaxis. N Engl J Med.

[5] Cohen MS, Shaw GM, McMichael AJ, Haynes BF (2011). Acute HIV-1 infection. N Engl J Med.

[6] McCormack S, Dunn DT, Desai M, Dolling DI, Gafos M, Gilson R (2016). Pre-exposure prophylaxis to prevent the acquisition of HIV-1 infection (PROUD): effectiveness results from the pilot phase of a pragmatic open-label randomised trial. Lancet.

